# A Systematic Review on PETTICOAT and STABILISE Techniques for the Management of Complicated Acute Type B Aortic Dissection

**DOI:** 10.31083/j.rcm2402034

**Published:** 2023-01-31

**Authors:** Petroula Nana, George Kouvelos, Christian-Alexander Behrendt, Athanasios Giannoukas, Tilo Kölbel, Konstantinos Spanos

**Affiliations:** ^1^Vascular Surgery Department, Larissa University Hospital, Faculty of Medicine, University of Thessaly, 41110 Larissa, Greece; ^2^German Aortic Center, Department of Vascular Medicine, University Heart and Vascular Center UKE Hamburg, 20251 Hamburg, Germany

**Keywords:** acute, dissection, type B, bare metal stent, stabilise, petticoat

## Abstract

**Background:**

Extended downstream endovascular management has been applied in acute complicated type B aortic dissection (acTBAD), distally to standard thoracic endovascular aortic repair (TEVAR), using bare metal stents, with or without lamina disruption, using balloon inflation. The aim of this systematic review was to assess technical success, 30-day mortality, and mortality during follow-up in patients with acTBAD managed with the Provisional Extension To Induce Complete Attachment (PETTICOAT) or stent-assisted balloon-induced intimal disruption and relamination (STABILISE) technique.

**Methods:**

The Preferred Reporting Items for Systematic Reviews and Meta-analyses (PRISMA) 2020 statement was followed. A search of the English literature, via Ovid, using MEDLINE, EMBASE, and CENTRAL databases, until 30th August 2022, was executed. Randomized controlled trials and observational studies (published between 2000–2022), with ≥5 patients, reporting on technical success, 30-day mortality and mortality during the available follow-up among patients that underwent PETTICOAT or STABILISE technique for acTBAD were eligible. The Newcastle-Ottawa Scale was applied to assess the risk of bias. Primary outcomes were technical success and 30-day mortality, and secondary outcome was mortality during the available follow-up.

**Results:**

Thirteen studies were considered eligible, twelve in the quantitative analysis. In total, 418 patients with acTBAD managed with the PETTICOAT (83%) or STABILISE (17%) technique were included. Technical success ranged between 97–100%, 99% for the PETTICOAT and 100% for the STABILISE sub-cohort. Thirty-day mortality was estimated at 3.7% (12/321), 1.4% for the STABILISE and 4.4% for the PETTICOAT technique. All studies reported the mean available follow-up which was estimated at 20 months (range 3–168 months), 22 months (mean value) for the PETTICOAT and 17 months (mean value) for the STABILISE technique. Twenty-three patients died during follow-up, with an estimated mortality rate at 5.7% for the total cohort. The mortality during follow-up was 0% for the STABILISE and 7.0% for the PETTICOAT approach.

**Conclusions:**

Both, the PETTICOAT and STABILISE techniques presented less than 4% perioperative mortality in patients with acTBAD with high technical success rate. The mid-term mortality rate was at 6%. However, the heterogeneity in the available studies’ highlights the need for further prospective studies, including larger volume and longer follow-up.

## 1. Introduction

Acute complicated type B aortic dissection (acTBAD) represents a potentially fatal aortic emergency, characterized by the incidence of rupture or impending rupture and/or malperfusion [1]. Malperfusion represents an end-organ ischemia due to static or hemodynamic obstruction [1]. Emergent intervention is indicated in acTBAD in contrast to uncomplicated TBAD, that can frequently be managed conservatively [1]. Current guidelines recommend endovascular management in acTBAD (Class I Level of evidence C) as a first line treatment while early endografting may be considered in selective uncomplicated cases prone to unfavourable evolvement [2]. Thoracic endovascular aortic repair (TEVAR) has shown reduced peri-operative mortality and acceptable survival, more than 63% at 3 years, in acute complicated and uncomplicated cases of TBAD, with comparable findings between groups [3, 4, 5].

The benefit of endovascular management in acTBAD is not restricted to short-term survival. TEVAR in acute TBAD improves aortic remodeling more favorable compared to chronic TBAD, preventing aneurysm formation and rupture risk [6, 7, 8, 9]. However, remodeling after TEVAR is usually limited to the thoracic aorta leaving the abdominal aorta dissected and at risk for aneurysmal dilatation [7]. Provisional Extension To Induce Complete Attachment (PETTICOAT) and Stent-assisted balloon-induced intimal disruption and relamination (STABILISE) techniques have been introduced to improve the outcomes of TEVAR in patients with acTBAD [10, 11].

The aim of this systematic review was to assess the technical success and 30-day mortality as well as follow-up outcomes in patients suffering from acTBAD, managed using the PETTICOAT or STABILISE technique.

## 2. Materials and Methods

### 2.1 Eligibility Criteria

The Preferred Reporting Items for Systematic Reviews and Meta-analyses (PRISMA) guidelines were followed [12]. Randomized controlled trials (RCTs) and prospective or retrospective observational studies, published between 2000 and 2022, of the English medical literature, reporting on technical success, 30-day mortality, and mortality during follow-up in patients with acTBAD managed with the PETTICOAT or STABILISE technique were eligible and incorporated in the current systematic review. Studies reporting on type A aortic dissection or subacute or chronic TBAD were not considered eligible. In case that a study reported mixed population findings, it was considered eligible, only if outcomes on acute cases could be safely extracted. Furthermore, studies reporting only on TEVAR outcomes, open or hybrid repair were excluded. Case reports and case series with less than 5 patients were also omitted.

### 2.2 Search Strategy

A systematic search via Ovid, of MEDLINE and EMBASE, and CENTRAL databases, was conducted with an endpoint set for August 31st, 2022. The PICO model [Patient; Intervention; Comparison; Outcome (**Supplementary Table 1**)] was applied [13]. The following search items, including Expanding Medical Subject Heading (MeSH terms), were used in various combinations (Table 1): (acute), (complicated), (dissection), (PETTICOAT), (STABILISE), (bare metal stent), (endovascular repair), (technical success), (mortality). Scrutiny was accomplished independently after full-text assessment by two investigators (P.N., K.S.) and discrepancies were resolved after discussion with a third investigator (T.K.).

**Table 1.** **Search strategy**.

Frame	Mesh terms	Search	Inclusion criteria	Exclusion criteria	Sources
P (patients, participants, population)	#1. #2. #3. #4. “Acute” AND “Complicated” AND “Dissection” AND “Type B”	#1. AND #2. AND #3. AND #4. AND #5. OR#6. OR #7. AND #8. AND #9. OR #10.	Randomized Controlled Trials and comparative observational studies, retrospective or prospective, reporting on technical success, 30-day mortality, and mortality during the available follow-up in patients with acute complicated type B aortic dissection managed with the STABILIZE or PETTICOAT technique Peer-review journals English language	Irrelevant title	Databases (Medline, EMBASE via OVID and Cochrane library)
				Irrelevant full text	
				Non-English	
				Editorial, reviews, meta-analyses, technical notes, images, case series <5 patients, case reports	
				Studies reporting on previously treated dissections, type A aortic dissection, subacute or chronic type B dissections, dissections of the infrarenal aorta, standard thoracic endovascular aortic repair or conventional open repair	
I (intervention)	#5. #6. #7. #8. “STABILIZE” OR “PETTICOAT” OR “Bare metal stent” AND “Endovascular”				
C (reference test)	NA				
O (outcome)	#9. #10. “Technical success” “Mortality”				
Time	Search period: 2000–2022				
	Last search: 31.08.2022				

### 2.3 Data Extraction

A Microsoft Excel (Office 365, Microsoft, Redmond, WA, USA) file was generated. Extracted data included study characteristics (authors, journal, date of publication or acceptance, study design, study period, country/center/database, aim) in addition to general information [demographics (age, sex), indication to treat (malperfusion, rupture/impending rupture), technique (PETTICOAT, STABILISE) and technical details (type of endograft, type of bare metal stent, distal extension, balloon, stenting of aortic branches, duration of operation)]. Technical success, mortality at 30-days and mortality during the available follow-up were recorded. Morbidity rupture, stent induced entry tear (SINE), retrograde dissection, endoleak type 1 (EL 1), renal insufficiency, malperfusion, cerebrovascular events (stroke and transient ischemic attack), spinal cord ischemia (SCI; paresis or paraplegia) at 30-days was recorded and analyzed. The available follow-up of each study was extracted when reported. The imaging method of surveillance, false lumen (FL) thrombosis rate of the thoracic and abdominal aorta, any remodeling data, including aortic diameter and volume, were assessed when available. Regarding follow-up outcomes, mortality, rupture, retrograde dissection, EL 1, re-intervention and open conversion were recorded and analyzed. Missing data assessment and funding information were also extracted when available. Regarding potential overlapping studies, the latest available data were included in the analysis.

### 2.4 Quality Assessment

The quality of the included studies was assessed with the Newcastle-Ottawa Scale (NOS, **Supplementary Table 2a**) while for the RCT the JADAD tool was used (**Supplementary Table 2b**) [14, 15]. NOS appraises three main methodological domains: selection methods, comparability on design or analysis, and assessment of outcomes. Individual studies were attributed a higher risk of bias in cases of inadequate confounder control and retrospective nature. Furthermore, any potential loss to follow-up or missing data that was not clearly stated in text were considered an additional confounder. The scale consists of a star system, with a maximum of nine stars. Studies achieving at least seven stars were characterized of higher quality [14]. JADAD is a multidisciplinary panel of six judges which are used to determine the effect of rater blinding on the assessments of quality. The final version of the instrument includes three items. These items were scored consistently by all the raters, as blind assessments produced significantly more consistent scores [15].

### 2.5 Outcomes

The primary outcomes were technical success and 30-day mortality in patients that underwent acTBAD management using the PETTICOAT or STABILISE technique. The mortality during the available follow-up was considered a secondary outcome.

### 2.6 Definitions

As there was a significant heterogeneity among studies, especially for anatomic modifications during follow-up, the definitions reported by each study are displayed in Table 2 (Ref. [10, 16, 17, 18, 19, 20, 21, 22, 23, 24, 25, 26, 27]).

**Table 2.** **Definitions of technical success and aortic remodelling provided by the included studies**.

Study	Definition for technical success	Definition for aortic remodelling
Hofferberth, *et al*. [16]		
Liu, *et al*. [17]	Complete sealing of the primary entry tear followed by obliteration of FL in at least the thoracic region	
Lombardi, *et al*. [18]		FL thrombosis partial or complete to thoracic aorta
He, *et al*. [19]	Endograft deployment without endoleak type I/III and absence of OSR or death within 24 h	TL re-expansion with concomitant complete thrombosis and retraction of the FL
Kische, *et al*. [20]		Complete FL thrombosis of thoracic aorta
Sobocinski, *et al*. [21]		Complete FL thrombosis of thoracic aorta
Faure, *et al*. [22]		Complete FL obliteration of thoracic aorta
Kahlberg, *et al*. [23]		FL thoracic aorta complete thrombosis or disappear
Lombardi, *et al*. [24]		FL thrombosis partial or complete to thoracic aorta
Lombardi, *et al*. [10]		FL partial or complete thrombosis
Kazimierczak, *et al*. [25]	Resolution of complications, sealing in proximal landing zone, relamination of dissecting lamella along thoracic grafts and iliac stents, visceral BMS-XL sufficiently dilated without complications; stopped FL perfusion in thoracic segment	Stable aortic size (max change <5 mm), complete TL expansion, complete FL thrombosis
Lin, *et al*. [26]	Complete exclusion of the primary entry without any complications	FL thoracic aorta complete thrombosis
Hsu, *et al*. [27]	Successful implantation of stent grafts and BMS without intraoperative endoleak type IA	FL thrombosis

Footnotes: BMS, bare metal stent; FL, false lumen; OSR, open surgical repair; TL, true lumen.

### 2.7 Statistical Analysis

Continuous data were reported as a mean ± standard deviation. Categorical data were expressed as absolute numbers with the associated range. The effect of measures for technical success, early and follow-up mortality, as well as the remaining outcomes were presented as percentages or proportions of the included studies for each outcome. For missing data, there was no imputation and the effect of measure of each outcome was estimated on the cohort of the studies reporting on each specific outcome. No comparison between the techniques was executed. Statistical analyses used SPSS 20.0 software (IBM Corp, Armonk, NY, USA).

## 3. Results

### 3.1 Qualitative and Quantitative Analysis 

**Fig. 1.**
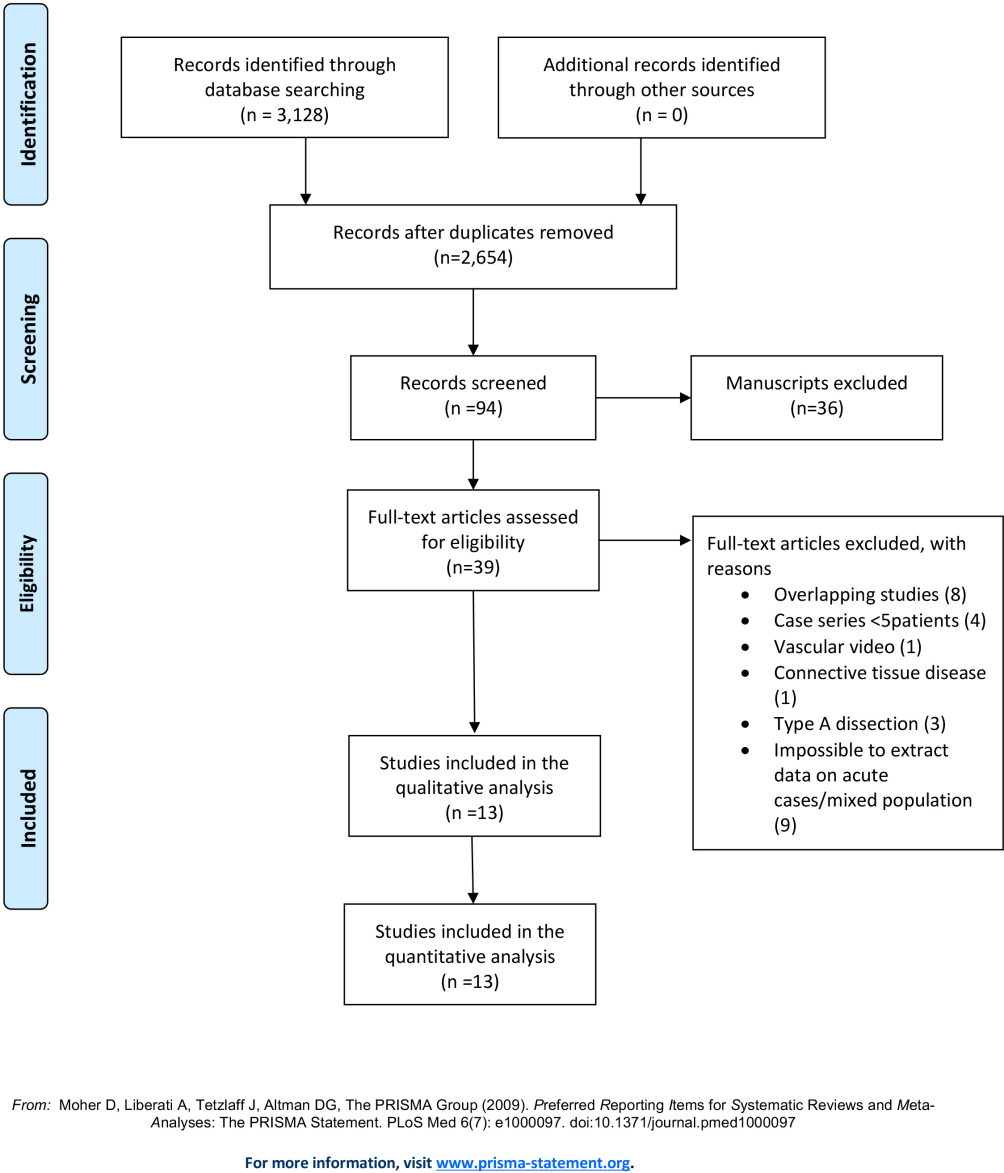
**PRISMA flow**. The initial search yielded 3128 articles. After exclusion of studies according to the reported criteria, thirteen studies were included in this systematic review.

The initial search yielded 3128 articles. Deduplication was performed automatically using Ovid (474 studies excluded). After exclusion of studies according to the previously reported criteria, thirteen studies were included in this systematic review (Fig. 1) [10, 16, 17, 18, 19, 20, 21, 22, 23, 24, 25, 26, 27]. Three studies were prospective observational studies while one was a randomized controlled trial [10, 18, 24, 26]. The remaining studies were retrospective. Regarding the study of Sobocinski* et al*. [21], only the anatomic modification data during follow-up were extracted and presented in this analysis in order to overcome any potential overlap with previously reported patients’ outcomes. In studies, reporting on acute, subacute, and chronic cases, only data regarding acTBAD were included [18, 21, 24, 27].

In total, 418 patients with acTBAD managed with the PETTICOAT (83%; 346/418) or STABILISE (17%; 72/413) techniques were included; 254 were males (81.4%, 254/312) [17, 18, 19, 22, 23, 24, 25, 26, 27]. The mean age was 56.0 ± 10.1 years [18, 19, 20, 23, 24, 25, 26, 27]. The indication for TEVAR was described in all studies. The specific indications, including true lumen collapse, FL expansion and persisting high flow to the FL per study are depicted in Table 3 (Ref. [10, 16, 17, 18, 19, 20, 21, 22, 23, 24, 25, 26, 27]), along with the studies’ main characteristics. Eleven studies reported outcomes regarding the PETTICOAT technique while two provided data on the STABILISE approach [10, 16, 17, 18, 19, 20, 21, 22, 23, 24, 25, 26, 27]. In one study, the extended PETTICOAT technique was applied [25].

**Table 3.** **Studies characteristics and indications to treat**.

Study	Year	Center/Country	Type of study	Study timespan	Study population	N of patients	Indication to treat	Malperfusion	Rapid progression	Rupture
Hofferberth *et al*. [16]	2012	Australia	Retrospective, observational cohort	2003–2010	Patients with acTBAD managed with STABLE procedure	16	Malperfusion, TL collapse			
Liu *et al*. [17]	2013	Japan, China	Retrospective observational cohort	2009–2011	Patients with cTBAD that underwent TEVAR & PETTICOAT	33	Malperfusion, imminent rupture, rupture, intractable chest pain, FL aneurysm formation, uncontrollable HT			
Lombardi *et al*. [18]	2014	Multicenter	Multicenter, prospective trial	2007–2012	Patients with cTBAD that underwent TEVAR & PETTICOAT	55	Malperfusion, impending rupture, resistant hypertension, persistent pain/symptoms, or aortic growth >5 mm within 3 months, transaortic diameter >40 mm	38	19	11
							PETTICOAT was performed if branch vessel obstruction or false lumen perfusion persisted			
He *et al*. [19]	2015	Changsha, China	Retrospective observational cohort	2010–2013	Patients with cTBAD that underwent TEVAR & PETTICOAT	35	Malperfusion, impending rupture, aortic expansion, hemothorax, resistant HT, persistent pain, and TL collapse			5
Kische *et al*. [20]	2015	Berlin, Germany	Retrospective observational cohort		Patients with cTBAD that underwent TEVAR & PETTICOAT	17	Malperfusion and incomplete TL expansion or high-flow FL	15		
Sobocinski *et al*. [21]	2016	France, Sweden	Retrospective analysis of prospective data, single center, observational cohort	2007–2012	Patients with cTBAD that underwent TEVAR & PETTICOAT	NA (includes STABLE I acute cases)	Malperfusion, impending rupture, resistant hypertension, persistent pain/symptoms, or aortic growth >5 mm within 3 months, transaortic diameter >40 mm, PETTICOAT was performed if branch vessel obstruction or false lumen perfusion persisted			
Faure *et al*. [22]	2018	Paris, France	Retrospective analysis of prospective data, single center, observational cohort	2011–2017	Patients with acute cTBAD that underwent TEVAR & PETTICOAT	41	Malperfusion or poor anatomic characteristics including >40 mm aorta	20	3	3
Kahlberg *et al*. [23]	2019	Milan, Italy	Retrospective observational cohort	2016–2019	Patients with acute cTBAD managed with STABILIZE	14	Malperfusion	9	3	
Lombardi *et al*. [24]	2019	Multicenter	Multicenter, prospective trial	2007–2012	Patients with cTBAD that underwent TEVAR & STABILIZE	55	Malperfusion, impending rupture, resistant hypertension, persistent pain/symptoms, or aortic growth >5 mm within 3 months, transaortic diameter >40 mm, PETTICOAT was performed if branch vessel obstruction or false lumen perfusion persisted			
Lombardi *et al*. [10]	2019	Multicenter	Multicenter, prospective trial	2012–2015	Patients with cTBAD that underwent TEVAR & PETTICOAT	73	Malperfusion, Rupture	57	17	20
Kazimierczak *et al*. [25]	2020	Poland	Retrospective analysis of prospective data, single center, observational cohort	2014–2015	Patients with acute cTBAD that underwent TEVAR & PETTICOAT	17	Malperfusion, Rupture	17		6
Lin *et al*. [26]	2020	China	Prospective, RCTs	2010–2013	Patients with DeBekay IIIB dissection that received extended PETTICOAT	42	Rapid aortic expansion (diameter ≥60 mm or expansion rate ≥10 mm in hospital), rupture and/or hypotension/shock, malperfusion, paraplegia/paraparesis; periaortic hematoma; recurrent or refractory pain; and/or refractory hypertension	16		4
Hsu *et al*. [27]	2021	Taiwan	Retrospective, comparative study	2005–2017	Patients with cTBAD that underwent TEVAR & PETTICOAT	20	Malperfusion syndrome, rupture/impending rupture, uncontrolled HT, persistent pain or high-risk radiographic features (pleural effusion, aorta >40 mm)			

Footnotes: cTBAD, complicated type B aortic dissection; HT, hypertension; N, number; RCT, randomized controlled trial; TEVAR, thoracic aortic endovascular repair; TL, true lumen.

Four studies reported specific anatomic preoperative characteristics, including aortic diameter and volume, as displayed in **Supplementary Table 3** (Ref. [19, 21, 25, 27]). In nine studies, left subclavian artery (LSA) management was reported in detail [10, 17, 19, 20, 21, 22, 23, 25, 27]. In one study, LSA occlusion related to upper limb ischemia was managed conservatively using alprostadil [17]. In the remaining studies, LSA revascularization was performed using conventional bypass from the left common carotid artery (44 cases), or the periscope technique (13 cases) [10, 19, 20, 21, 22, 23, 25, 27].

Regarding the type of bare metal stent, in seven studies the Zenith Dissection Endovascular System (Cook Medical, Bloomington, IN, USA) was used [10, 16, 18, 22, 24, 27]. A combination of the Zenith Dissection Endovascular System and Zenith TX2 endograft for proximal coverage was reported in six of them [10, 16, 18, 24, 27]. In the remaining studies a variety of devices has been used, as depicted in Table 4 (Ref. [10, 16, 17, 18, 19, 20, 21, 22, 23, 24, 25, 26, 27]), along with technical details reported in each study. The reported oversizing of the endograft ranged between 0–15% while the oversizing of the bare metal stent was 0–25% [18, 20, 21, 23, 24, 26]. Three studies provided data on the STABILISE technique and reported the use of a molding balloon with diameter 26 mm–42 mm or 46 mm, dilated up to 2–4 atmospheres , to achieve lamina disruption and stabilization [22, 23, 25].

**Table 4.** **Technical details, as stent and bare metal stent type, reported in the included studies**.

Study	Type of graft	N of grafts	Oversizing of endograft	Length of covered aorta by grafts	Type of BMS	Overlap between graft and BMS	Oversizing of BMS	Length of BMS	Balloon	Balloning of BMS
Hofferberth *et al*. [16]	Zenith Dissection Endovascular System (TXD Systems, William Cook Europe, Bjaeverskov, Denmark)									
Liu *et al*. [17]	Valiant (Medtronic, Santa Rosa, CA, USA)	BMS deployed initially at intended distal landing site of the stent-graft in TL	15%	157.4 (120–200)	Sinus-XL; OptiMed, Ettlingen, Germany		15%	72.7 (60–80)		
Lombardi *et al*. [18]	Zenith TX2 TAA Endovascular Graft with Pro-Form (Cook Medical, Bloomington, Ind, USA)				Zenith Dissection Endovascular System (Cook Medical, Bloomington, Ind, USA)					
He *et al*. [19]	10 Zenith TX2 (Cook Medical, Bloomington, Ind, USA), 2 Relay (Boston Scientific Corporation, Marlborough, MA, USA), 18 Hercules (Microport, Shanghai, China), 5 Talent (Medtronic, Santa Rosa, CA, USA)			152.4 (120–200)	35 Sinus-XL stent, (OptiMed, Ettlingen, Germany)	3–4 cm	0% of TL	70.7 (60–80)		
Kische *et al*. [20]			10%		18 Zenith (Cook Medical, Bloomington, Ind, USA), 1 Fortress	3–4 cm	0–25%	170.2 ± 50.8		
Sobocinski *et al*. [21]										
Faure *et al*. [22]	34 CTAG, Gore, 3 TX2 Zenith (Cook Medical, Bloomington, Ind, USA), 4 Relay PluS (Boston Scientific Corporation, Marlborough, MA, USA)			200 (150–300)	Zenith Dissection Endovascular System (Cook Medical, Bloomington, Ind, USA)		20%	185	26–42 trilobe Gore Medical, Flagstaff, AZ, USA	dilation with 1–2 atm mannually to disrupt lamina down to the infra-renal aorta
Kahlberg *et al*. [23]	13 Zenith TX2 & Alpha (Cook Medical, Bloomington, Ind, USA), 1 cTAG		10%				0% for TL + FL		46	
Lombardi *et al*. [24]	Zenith TX2 TAA Endovascular Graft with Pro-Form (Cook Medical, Bloomington, Ind, USA)				Zenith Dissection Endovascular System (Cook Medical, Bloomington, Ind, USA)					
Lombardi *et al*. [10]	Zenith TX2 TAA Endovascular Graft with Pro-Form (Cook Medical, Bloomington, Ind, USA)	1 to 3			Zenith Dissection Endovascular System (Cook Medical, Bloomington, Ind, USA)					
Kazimierczak *et al*. [25]	Valiant, (Medtronic, Santa Rosa, CA, USA)	One proximal to cover entry tear and a second to cover the descending aorta up to 5cm before CT	Proximal 10% for TL + FL diameter, distal 10–15% for TL + FL		200 mm BMS XL (Medicut, Pforzheim, Germany)	5 cm	0% for TL + FL		46, Reliant, Medtronic	Dilation did not exceed total aortic diameter inside the BMS to avoid rupture
Lin *et al*. [26]	22 Endurant (Medtronic Cardiovascular, Santa Rosa, CA, USA), 14 Ankura (LifeTech Scientific, Shenzhen, China), 4 Zenith TX2 (William A. Cook Australia, Brisbane, Australia), 2 Hercules (MicroPort, Shanghai, China)		0–15%	178.6 ± 25.0	Wallstent (Boston Scientific Corporation, Marlborough, MA, USA)	2–4 cm	0% max diameter of TL	70		
Hsu *et al*. [27]	Zenith Dissection Endovascular System (Cook Inc, Bloomington, Ind, USA)			136.1 ± 21.0	Zenith Dissection Endovascular System (Cook Medical, Bloomington, IN, USA)			221.0 ± 41.1		

Footnotes: BMS, bare metal stent; CT, celiac trunk; FL, false lumen; N, number; TL, true lumen.

Three studies reported further management of aortic branches with the application of additional stenting when indicated, as in case of persistent malperfusion or dissection extension [22, 23, 25]. In total, 30 stents were deployed into provisionally selected target vessels [22, 23, 25]. One analysis reported the use of extended PETTICOAT with common iliac artery endograft deployment (Endurant, Medtronic, Santa Anna, CA, USA) [25]. In this study, the endograft limbs were extended into the aortic bare metal stent up to renal arteries, as kissing limbs [25]. Two studies reported the use of self-expanding stents for external iliac artery coverage, in one of them, covered stents were selected [23, 25].

Regarding the intra-operative details, only four studies provided data on operation time while two of them also reported the fluoroscopy duration and three, the contrast volume [10, 17, 19, 20]. The estimated duration of operation was 112 minutes (range 54–519 minutes) [10, 17, 19, 20]. The fluoroscopy time was 13.5 minutes (median; range 11–17 minutes) in one study and 27 ± 7 minutes in the second one [17, 19]. Contrast volume was estimated at 238 mL (range 89–400 mL) [17, 19, 20].

### 3.2 Early Outcomes

Technical success was stated in six studies, three of them reported on the STABILISE technique [17, 19, 22, 23, 25, 27]. The estimated technical success rate was 99.5% (range 97.1–100%), 99% for the PETTICOAT and 100% for the STABILISE cohort [22, 23, 25].

Mortality at 30-days was reported in ten studies [10, 16, 17, 18, 19, 20, 22, 23, 25, 27]. In total, twelve deaths were recorded with an estimated 30-day mortality of 3.7% (12/321). Three deaths were related to aortic rupture, leading to an estimated early aorta-related death rate of 1.2% (3/247) [10, 17, 18, 19, 23, 25, 27]. When the cohort was separated into subgroups, the 30-day mortality was 1.4% (1/72) for the STABILISE technique [22, 23, 25], and 4.4% for the PETTICOAT approach (11/249) [10, 16, 17, 18, 19, 20, 27]. All aortic ruptures were reported in the PETTICOAT subgroup, leading to an early aorta-related death rate of 1.4% (3/216) [10, 17, 18, 19, 27].

SINE was recorded in four studies, two of them reporting on the STABILISE technique [17, 23, 25, 27]. Two events were reported, both patients were managed with the PETTICOAT technique [total cohort rate: 2.3% (2/84), PETTICOAT rate: 3.8% (2/57)] [17, 27]. Three cases of retrograde type A aortic dissection were extracted from studies, all belonged to the PETTICOAT group [total cohort rate: 1.6% (3/183), PETTICOAT rate: 2.3% (3/128)].

Regarding post-operative morbidity, 17 cases of renal insufficiency were reported, all in the PETTICOAT cohort [total cohort rate: 6.3% (17/268), PETTICOAT rate: 8.7% (17/196), STABILISE rate: 0% (0/72)] [10, 17, 18, 19, 22, 23, 25]. Regarding cerebrovascular events, 14 adverse events were recorded, two of them in the STABILISE group with a rate of 4.9% (14/288) for the total cohort and, 5.6% (12/216) and 2.8% (2/72) for the PETTICOAT and STABILISE techniques, respectively [10, 17, 18, 19, 22, 23, 25, 27]. SCI was reported in eleven cases (4.3%, 11/253), five of them were characterized as paresis (2.8%, 5/180) and two as paraplegias (0.9%, 2/215) [10, 17, 18, 19, 22, 23, 25, 27]. Among the SCI events, four were recorded to the STABILISE cohort (5.6%, 4/72) and the remaining to the PETTICOAT group (3.9%, 7/181) [10, 17, 18, 19, 22, 23, 25, 27].

### 3.3 Follow-up Findings

All studies reported on the available follow-up which was estimated at 20 months (range 3–168 months) [10, 16, 17, 18, 19, 20, 21, 22, 23, 24, 25, 26, 27]. For the studies reporting on the PETTICOAT technique the estimated follow-up was 22 months (3–40 months) and for the STABILISE technique, it was 17 months (1–168 months). Regarding mortality, 23 patients died during the available follow-up: 7 cases were reported as aorta-related and 4 ruptures were reported [10, 17, 18, 19, 20, 22, 23, 24, 25, 26, 27]. The estimated mortality rate was 5.7% (23/402), with an aorta-related mortality rate at 33% (7/21) for the total cohort [10, 18, 19, 22, 23, 24, 26]. When the cohort was separated into subgroups, the mortality during follow-up was 0% (0/72) for the STABILISE technique [22, 23, 25], and 7.0% for the PETTICOAT approach (23/330) [10, 18, 19, 22, 23, 24, 26]. All aorta-related deaths and ruptures were recorded among patients that were managed with the PETTICOAT technique.

Regarding post-operative adverse events, eleven cases of retrograde type A aortic dissection were reported [10, 16, 18, 19, 22, 23, 24]. The rate for the total cohort was estimated at 3.8% (11/289), for the PETTICOAT group it was 4.3% (10/234) and for the STABILISE, 1.8% (1/55). endoleak type 1A (EL 1A) was reported in eight studies [10, 17, 22, 23, 24, 25, 26, 27]. Twelve endoleaks 1A (ELs 1A) were recorded leading to 3.7% rate (11/295) for the total cohort. Re-interventions were reported in seven studies while the estimated reinterventione rate during follow-up was 10.1% (32/315) [10, 18, 19, 22, 23, 24, 26]. Twenty-three events were detected in the PETTICOAT group (8.8%, 23/260) and nine in the STABILISE group (16.3%, 9/55). Among them one open conversion has been recorded in the study published by Lombardi *et al*. [24].

As a significant heterogeneity in definitions and assessment of anatomic aortic modifications after TEVAR was detected in the included studies. The anatomic modifications are displayed in Table 5 (Ref. [10, 16, 17, 18, 19, 20, 21, 22, 23, 24, 25, 26, 27]), as presented by the included studies. Studies reported a favourable remodelling (expansion of true lumen and total aortic diameter stabilization compared to pre-operative aortic diameters) ranging from 17.6 to 100% for the thoracic aorta [17, 18, 19, 20, 21, 22, 23, 24, 25, 26, 27]. In two studies, a true lumen expansion was detected during follow-up compared to the pre-operative estimation [20, 26].

**Table 5.** **Anatomic details of the aorta after the application of the PETTICOAT and STABILISE techniques**.

Study	Favorable remodelling	Thoracic or total aorta	FLV	TLV	AV	TL or aortic lumen	Arch diam	LSA diam	Descending aortic diam	CT diam	SMA diam	LRA diam	Infra-renal diam
Hofferberth *et al*. [16]													
Liu *et al*. [17]	100%												
Lombardi *et al*. [18]	85.1%												
He *et al*. [19]	76.5%	Total	108 ± 54	227 ± 43	335 ± 97								
Kische *et al*. [20]	17.6%					TL	31.3 ± 2.0	30.6 ± 3.3		21.5 ± 4.5	20.4 ± 4.4		
Sobocinski *et al*. [21]	38%	Thoracic	129 ± 12	230 ± 9	359 ± 16								
Faure *et al*. [22]	100%												
Kahlberg *et al*. [23]	93%												
Lombardi *et al*. [24]	74.1%												
Lombardi *et al*. [10]	78.3%												
Kazimierczak *et al*. [25]	100%	Total	72.6 ± 59	279 ± 105	338 ± 139	AL	35 ± 4.3	37 ± 5.2	41 ± 4.5	33 ± 5.6	33 ± 3.1	30 ± 4.9	30 ± 4.9
Lin *et al*. [26]	80.9%					TL			33.4 ± 2.5	20.3 ± 3.2			
Hsu *et al*. [27]	70%	Total	77.5 ± 24.7	171.1 ± 9.6	248.6 ± 34.3								

Footnotes: AV, aortic volume; CT, celiac trunk; diam, diameter; FLV, false lumen volume; LRA, lower renal artery; LSA, left subclavian artery; SMA, superior mesenteric artery; TL, true lumen; TLV, true lumen volume.

### 3.4 Risk of Bias

Five out of thirteen studies were considered of high quality (>7 stars). The remaining were characterized as low quality (61.5%), due to small number of cases, surgeon, and patients’ selection according to surgeons’ experience and patients’ anatomic characteristics, and limited follow-up and missing data. Regarding the RCT, the application of JADAD demonstrated a moderate quality.

## 4. Discussion

This systematic review suggests that both, the PETTICOAT and STABILISE techniques, can be applied in patients managed for acTBAD. In all studies the indication for treatment was either malperfusion, rupture or imaging findings related to high-risk for complications as recommended by current guidelines [1, 10, 16, 17, 18, 19, 20, 21, 22, 23, 24, 25, 26, 27]. Published experience is limited to less than 500 cases of acTBAD and available follow-up is limited to less than 2 years [10, 16, 17, 18, 19, 20, 21, 22, 23, 24, 25, 26, 27]. Despite the limited data, this systematic review detected an encouraging initial experience with high technical success, more than 99% and low early mortality. Endovascular management has developed over the years from standard TEVAR to PETTICOAT and STABILISE with an increasing number of patients that might benefit from an early intervention [23].

Thirty-day mortality in this systematic review was low, at 3.7% for the total cohort and up to 4.4% for the PETTICOAT technique. When considering that standard TEVAR for acTBAD has been reported with a 30-day mortality up to 5%, it seems that both techniques can be safely used as additional measures, without significant effect on patients’ early survival [3, 4]. Despite that the purpose of PETTICOAT and STABILISE is to provide a reduce distal stent-induced dissection rate and better aortic remodeling through years, the safety of both techniques in acTBAD remains of major importance [23, 24]. The lower mortality of the STABILISE technique may be related to the retrospective nature of the studies, as well as the limited number of cases available in the current literature. Series reporting on PETTICOAT for complicated TBAD, including acute and subacute cases, have shown that the addition of bare metal stents distally is related to less true lumen collapse and visceral malperfusion, with a 30-day mortality under 5% while similar findings have been reported for the STABILISE approach, despite the potential risk of intraoperative aortic rupture [7, 19].

Post-operative morbidity remained within acceptable rates, at 4% for SCI and 6.3% for renal insufficiency, while stroke rate was less than 5%, regardless that patients required more proximal landing-zones and additional debranching [10, 19, 20, 21, 22, 23, 25, 27, 28]. These findings are in accordance with the available literature regarding the use of standard TEVAR in acTBAD, where the estimated rates are at 5.8% for stroke and more than 7% for renal failure [3]. Potentially, the use of PETTICOAT and STABILISE technique, with the restoration of flow to the true lumen, associated to a provisionally aortic branch stenting, might have a positive impact on flow to aortic sidebranches [10]. The use of a limited coverage and the application of bare metal stents to the remaining aorta may also have a protective impact on SCI evolution [10].

TEVAR has been related to promising long-term outcomes in cases with acTBAD [29]. Especially when considering that the mean age of the reported cohorts with TBAD was below 60 years, the long-term survival is very relevant [29]. In this review, mortality at mid-term follow-up was less than 7% for either technique. However, in 30% of patients that died during the post*-*operative surveillance period, an aorta*-*related cause was reported, highlighting the fact that even with the application of more extensive treatment, long*-*term safety cannot be guaranteed [30]. Aortic rupture and retrograde type A aortic dissections are devastating complications after endovascular treatment for acTBAD and are related to post*-*operative fatal events with a mortality rate at 40% [31, 32].

Re-interventions are a major drawback of endovascular aortic repair. In this analysis, the rate was 10% during follow-up, and up to 16% for the STABILISE approach. However, only one open conversion has been recorded [10]. Studies including standard TEVAR cases have reported rates exceeding 20%, while acute TBAD management has been related to higher reintervention rates compared to a delayed endovascular treatment [7, 33, 34]. The re-intervention rate after extended endovascular management, was within acceptable rates. Disease evolution may be related to factors not associated to the extent of the aortic coverage and further interventions may be needed to improve results [35]. Patients and physicians should be aware that an extensive management does not exclude future secondary interventions and a specific follow-up protocol seems mandatory for the prevention of long-term complications [36].

Finally, aortic remodeling after extended aortic endovascular management in TBAD seemed to be improved using the reported techniques [10, 17, 18, 19, 20, 21, 22, 23, 24]. However, the lack of conformity in methodologic aspects does not permit an extended evaluation and summary of these findings. Sobocinski *et al*. [21, 37] concluded that PETTICOAT was related to significant thoracic true lumen expansion and FL regression rates during the initial 12 months of follow-up similar to standard TEVAR in TBAD. The favorable evolution of the thoracic aorta is not followed by a similar remodelling of the abdominal aorta [20, 21]. Follow-up data have shown that the total volume and especially, at the level of the abdominal aorta, continues to expand post-operatively, introducing an increased need for secondary interventions [38]. Additonally, SINE rate was estimated at 2.3% for the PETTICOAT technique, highlighting that despite that extended endovascular acTBAD management, this complication continues to happen [17, 26].

The number of cases managed with the PETTICOAT and STABILISE techniques continues to increase, 4 studies and 54 acute cases reported by 2014 and more than 400 cases and 14 studies by 2022 [39, 40]. However, thefindings of the current analysis should be interpreted cautiously in view of the low number of reported cases with acTBAD that are available in the currentl literature [17, 18, 19, 22, 23, 24, 25, 26, 27]. Despite that compared to previously published data, almost a decade ago, the number of acute cases managed with the PETTICOAT and STABILISE techniques continues to increase, the problematic arising in the literature, especially regarding the estimation of aortic remodeling and the variable results presented in limited studies, do not seem to be resolved [39, 40]. Further analyses, with more consistency in definitions and methods and longer follow-up, are needed to understand the long-term impact of PETTICOAT and STABILISE techniques in acTBAD.

### Limitations 

The retrospective nature of most of the included studies introduced certain bias and residual confounders. Studies reporting only on acute cases of TBAD and the use of PETTICOAT and STABILISE techniques were included a priori in this analysis. Thus, further details on both techniques and in other pathologic conditions are missing. The risk of bias varied considerably among studies. Furthermore, technical success, specific patient selection criteria, materials used, and follow-up data were not available in all studies. Variable procedures were performed, including additional stenting of the aortic branches and iliac arteries, that may have affected the potential outcomes, including clinical and anatomic findings. Especially for the PETTICOAT technique, the length and type of the deployed bare metal stents was under-reported and varied among studies. This fact potentially affected the outcomes of the included studies, and further, the findings of the current review. Regarding specific definitions, the heterogeneity was significant among studies, especially when considering true lumen collapse as an indication for repair and further, the methodologic assessment of aortic remodelling from the pre-operative to follow-up setting. Different approaches, including diameter and volumetric analyses, as well as estimation of them in variate anatomic positions did not permit a further estimation of the impact of PETTICOAT and STABILISE in aortic remodelling. Along these lines, a mathematical analysis could not be executed. As case reports and small case series were excluded, the findings of this analysis might have been affected. A meta-analysis could not be excecuted due to lacking comparative data between the techniques. The available follow-up was restricted to 20 months and long-term data are lacking from the literature.

## 5. Conclusions

Both, the PETTICOAT and STABILISE techniques presented less than 4% perioperative mortality in patients with acTBAD with high technical success rate. The mid-term mortality rate was at 6%. However, the heterogeneity in the available studies’ methodology does not permit firm conclusions. Further prospective analyses, including larger volume data and longer follow-up, are needed.

## Supplementary Material

Supplementary material associated with this article can be found, in the online version, at https://doi.org/10.31083/j.rcm2402034.
