# Supplementary material for: A Systematic Review on PETTICOAT and STABILISE Techniques for the Management of Complicated Acute Type B Aortic Dissection
Source: Rev Cardiovasc Med. 2023 Jan 31;24(2):34. doi: 10.31083/j.rcm2402034 (PMC11273109; doi:10.31083/j.rcm2402034)
Supplement: Supplementary file 1 [file 2153-8174-24-2-034-s1.zip › Supplementary Table.docx]

Supplementary Table 1.

| **P** | Patient, population, or problem | Patients with acute complicated type B aortic dissection |
| --- | --- | --- |
| **I** | Intervention, prognostic factor, or exposure | Endovascular management with the use of PETTICOAT and STABILIZE techniques |
| **C** | Comparison of intervention | Non-applicable |
| **O** | Outcome you would like to measure or achieve | Technical success, 30-day mortality, survival at 1 and 5 years |
|  | What type of question are you asking? | What is the estimated technical success and 30-day mortality in patients with acute cTBAD undergoing management with the use of PETTICOAT and STABILIZE techniques?  What is the estimated mortality during follow-up in patients with acute cTBAD undergoing management with the use of PETTICOAT and STABILIZE techniques? |
|  | Type of study you want to find | Randomized Controlled Trials, Observational studies, prospective or retrospective, reporting on technical success, 30-day mortality, and mortality during follow-up in patients with acute cTBAD undergoing management with the use of PETTICOAT and STABILIZE techniques? |

cTBAD, complicated Type B aortic dissection; PETTICOAT, Provisional Extension To Induce Complete Attachment; STABILIZE, Stent-assisted balloon-induced intimal disruption and re-lamination.

Supplementary Table 2.

| **Panel a.** | | | | | |
| --- | --- | --- | --- | --- | --- |
| **Studies** | **Year of publication** | **Selection** | **Comparability** | **Outcome** |  |
| Hofferberth, *et al* [16] | 2012 | *** | * | *** | 7 |
| Liu, *et al* [17] | 2013 | **** | ** | ** | 8 |
| Lombardi, *et al* [18] | 2014 | *** | * | *** | 7 |
| He, *et al* [19] | 2015 | **** | ** | ** | 8 |
| Kische, *et al* [20] | 2015 | **** | * | ** | 7 |
| Sobocinski, *et al* [21] | 2016 | *** | * | ** | 6 |
| Faure, *et al* [22] | 2018 | **** | ** | ** | 7 |
| Kahlberg, *et al* [23] | 2019 | **** | ** | ** | 8 |
| Lombardi, *et al* []24] | 2019 | **** | * | *** | 8 |
| Lombardi, *et al* [10] | 2019 | *** | * | *** | 7 |
| Kazimierczak, *et al* [25] | 2020 | **** | ** | ** | 8 |
| Hsu, *et al* [27] | 2021 | *** | ** | ** | 7 |

| **Panel b.** | |
| --- | --- |
| **Jadad criteria** | Lin, *et al* [26] 2020 |
| Described as randomized* | 1 |
| Described as double-blind* | 0 |
| Description of withdrawals | 1 |
| Randomization method described and appropriate** | 1 |
| Double blinding method described and appropriate** | 0 |
| **Score** | 3 |

* A study receives a score of 1 for "yes" and 0 for "no"

**A study receives a score of 0 if no description is given, 1 if the method is described and appropriate, and -1 if the method is described and inappropriate

Supplementary Table 3.

| Study | FL Volume | TL Volume | Aortic volume | Diameter of total aorta or TL | Arch diameter | LSA diameter | Discending aorta | CT diameter | SMA diameter | LRA diameter | Infra-renal diameter |
| --- | --- | --- | --- | --- | --- | --- | --- | --- | --- | --- | --- |
| Hofferberth, *et al* [16] |  |  |  |  |  |  |  |  |  |  |  |
| Liu, *et al* [17] |  |  |  |  |  |  |  |  |  |  |  |
| Lombardi, *et al* [18] |  |  |  |  |  |  |  |  |  |  |  |
| He, *et al* [19] | 319 ± 57 | 86 ± 38 | 405±95 | TL | 30.3 ± 1.6 | 16.7 ± 8.9 | 10.0 ± 3.5 |  |  |  | 9.4 ± 5.3 |
| Kische, *et al* [20] |  |  |  |  |  |  |  |  |  |  |  |
| Sobocinski, *et al* [21] | 203.1 ± 71.6 | 96.5 ± 38.3 | 296.6 ± 84.2 | Total aorta |  |  | 40.2 ± 5.7 |  |  |  | 33.2 ± 6.6 |
| Faure, *et al* [22] |  |  |  |  |  |  |  |  |  |  |  |
| Kahlberg, *et al* [23] |  |  |  |  |  |  |  |  |  |  |  |
| Lombardi, *et al* [24] |  |  |  |  |  |  |  |  |  |  |  |
| Lombardi, *et al* [10] |  |  |  |  |  |  |  |  |  |  |  |
| Kazimierczak, *et al* [25] | 229 ± 101 | 127 ± 90 | 356 ± 156 | Total aorta | 35 ± 4.4 | 37 ± 4.6 | 39±5.5 | 32 ± 3.1 | 31 ± 2.9 | 27 ± 4.2 | 27 ± 4.2 |
| Lin, *et al* [26] |  |  |  | TL |  |  | 13.8±2.6 | 11.5 ± 4.8 |  |  |  |
| Hsu, *et al* [27] | 164.2 ± 20.3 | 93.0 ± 8.0 | 157.2±28.3 | Total aorta |  | 34.9 ± 8.4 | 38.8±6.45 |  |  |  | 30.3 ± 16.5 |
